# Decision-Tree Based Model Analysis for Efficient Identification of Parameter Relations Leading to Different Signaling States

**DOI:** 10.1371/journal.pone.0082593

**Published:** 2013-12-18

**Authors:** Yvonne Koch, Thomas Wolf, Peter K. Sorger, Roland Eils, Benedikt Brors

**Affiliations:** 1 Division of Theoretical Bioinformatics, German Cancer Research Center (DKFZ), Im Neuenheimer Feld 580, Heidelberg, Germany; 2 Institute of General Pathology, Heidelberg University Medical School, University of Heidelberg, Im Neuenheimer Feld 224, Heidelberg, Germany; 3 Department of Systems Biology, Harvard Medical School, 200 Longwood Avenue, Boston, Massachusetts, United States of America; 4 Department for Bioinformatics and Functional Genomics, Institute for Pharmacy and Molecular Biotechnology, and Bioquant Center, University of Heidelberg, Im Neuenheimer Feld 267, Heidelberg, Germany; Universitat Pompeu Fabra, Spain

## Abstract

In systems biology, a mathematical description of signal transduction processes is used to gain a more detailed mechanistic understanding of cellular signaling networks. Such models typically depend on a number of parameters that have different influence on the model behavior. Local sensitivity analysis is able to identify parameters that have the largest effect on signaling strength. Bifurcation analysis shows on which parameters a qualitative model response depends. Most methods for model analysis are intrinsically univariate. They typically cannot consider combinations of parameters since the search space for such analysis would be too large. This limitation is important since activation of a signaling pathway often relies on multiple rather than on single factors. Here, we present a novel method for model analysis that overcomes this limitation. As input to a model defined by a system of ordinary differential equations, we consider parameters for initial chemical species concentrations. The model is used to simulate the system response, which is then classified into pre-defined classes (e.g., active or not active). This is combined with a scan of the parameter space. Parameter sets leading to a certain system response are subjected to a decision tree algorithm, which learns conditions that lead to this response. We compare our method to two alternative multivariate approaches to model analysis: analytical solution for steady states combined with a parameter scan, and direct Lyapunov exponent (DLE) analysis. We use three previously published models including a model for EGF receptor internalization and two apoptosis models to demonstrate the power of our approach. Our method reproduces critical parameter relations previously obtained by both steady-state and DLE analysis while being more generally applicable and substantially less computationally expensive. The method can be used as a general tool to predict multivariate control strategies for pathway activation and to suggest strategies for drug intervention.

## Introduction

Cellular processes such as cell death, proliferation or differentiation highly depend on activities of signaling proteins. These interact in an orchestrated fashion, as pathways, to regulate a certain physiological outcome or function. Signaling processes can be modeled by systems of ordinary differential equations (ODEs). Model analysis aims to elucidate key features of the system, like sensitivity to certain parameters or switch-like behavior. Computational model analysis aims at quantitatively or qualitatively describing the system response to stimulation and at identifying conditions that control a cellular function.

Local sensitivity analysis determines the change in model output when single model parameters are varied and in that way identifies most influential factors [6], [12], [16]. Control analysis [11], a non-dynamical method, similarly identifies the most important reactions or proteins. Qualitative methods for model analysis consider certain steady states of the system as model output and assign these equilibria to distinct cellular states or phenotypes like death or survival of a cell [4], [5], [9], [15]. For small models, it is possible to analytically find conditions that lead to attraction to certain steady states [21]. More complex models, however, require computational methods to analyze a system for its qualitative response. They test whether variation in single parameters or in stimulation patterns leads to changes with regard to steady state or stability behavior [5], [9], [15], [17].

Many qualitative and quantitative methods for model analysis are univariate, i.e. the change in system behavior or response is analyzed with respect to variation of one parameter at a time. Thus, these methods can only identify single parameters as important factors for a system response. They are not suited to reveal multi-parametric control strategies of a system. Certain interaction patterns [14], like competition of activating and inhibiting factors for a common binding site, cannot be simply broken down to a chain of single-parameter influences. Cell fate decision is brought about by a reaction system rather than by the influence of a single molecule [15], [20], [25]. Hence, a multivariate method for model analysis would be more adequate than a univariate approach. It should take into account simultaneous changes of several parameters resulting in a particular system response and hence identify strategies involving several co-operating species instead of a single perturbation to cause a certain system outcome.

We present here an automated, multivariate method that identifies conditions of parameter combinations. ODE systems use rate constants and initial concentrations of molecular species as parameters. While rate constants are believed to be constant at defined temperature, initial concentrations may vary considerably between different cell types and developmental or cell cycle stages [22]. Rate constants may be taken from literature, be inferred from direct measurement, or can be estimated by model fitting to time course data on concentrations of species of the system. If not known (or only up to a certain order of magnitude), they could be varied similarly to initial concentrations. Our approach, however, focusses only on initial concentrations in an ODE-system while keeping the rate constants of a given model fixed.

One example on the importance of initial concentrations is CD95-mediated apoptosis, where a threshold mechanism was identified to decide on cell fate [6], [18]. In this case, the ratio of molecules competing for the same binding site was found to influence apoptosis. In order to account for influences of molecule *ratios*, we use relations of initial concentrations instead of absolute values as input to the algorithm. We first categorize the system response according to pre-defined criteria. We use the model to simulate the dynamic behavior of the system with randomly created parameter sets for initial concentrations. Parameter sets are drawn from parameter ranges which are set based on prior biological knowledge. We then use decision trees as learning algorithms to find patterns in the parameter sets that led to a particular system response. The results of our method are expressed in simple rules containing relations with regard to molecule abundance (e.g. [species A] < [species B]) illustrated by an intuitively understandable decision tree representation, or rule set [10]. As a proof of concept, we have applied our method to a model of EGF receptor internalization, which has been previously analyzed by analytical solving and systematic parameter scan with respect to conditions leading to clathrin-independent internalization (CIE) [21]. To compare our method with a different multivariate approach, we further analyzed a caspase activation model which was formulated and examined by Aldridge et al. using direct Lyapunov exponent analysis [3]. Finally, we applied our method to a comprehensive apoptosis model by Albeck et al. incorporating both the (TRAIL-mediated) extrinsic as well as the intrinsic apoptosis pathway [2]. We will show that our method in all cases was able to reproduce well known parameter relations leading to certain cellular phenotypes at considerably less computational costs as compared to other multi-variate model analysis methods.

## Methods

**Figure 1:**
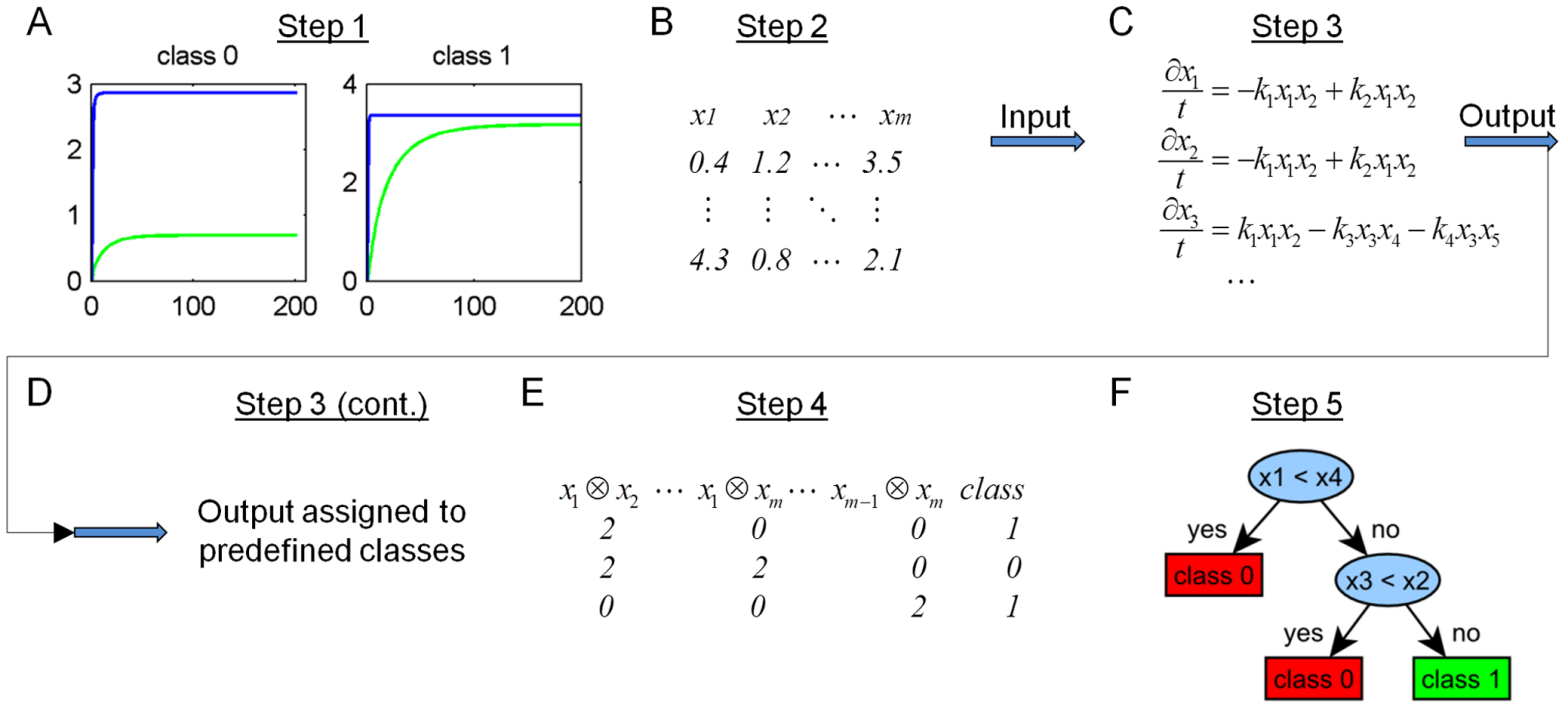
Workflow of multivariate model analysis. The presented approach for multivariate model analysis contains five steps: **A**) As a first step, response classes for the model outcome are defined. B) Secondly, a matrix of random initial values for model species having non-zero start values is generated. **C**) Initial values generated in step 2 are used in step 3 as input to the model of ODEs. **D**) Resulting output trajectories of interest are then classified according to the predefined criteria in step 1. **E**) In step 4, a matrix of species relations is constructed based on the matrix of initial concentrations generated in step 2. Pair-wise ratios of initial concentrations of molecule species A and B are encoded as ‘0’ in case a_0_ < b_0_, as ‘1’ in case a_0_  =  b_0_ and as ‘2’ in case a_0_ > b_0_, with a_0_ (b_0_) being the initial concentration of A (B). **F**) The matrix of species relations (step 4) together with the class information obtained from step 3 are then subjected as training data to a decision tree algorithm, which yields a tree representation on molecule relations that lead to the system response as defined in step 1.

Our method aims at providing insight into central multi-parametric control mechanisms of a model. This is done by a Monte Carlo approach, which involves random parameter generation and model simulation combined with a decision tree algorithm. Our approach assumes an existing differential equation-based model with given reaction rates, either obtained from model fitting or from biochemical experiments. We randomly generate initial concentrations within biologically reasonable parameter ranges, which are then used to simulate the system response by means of the ODE-based model. We then apply a decision tree algorithm to find patterns in the randomly sampled parameter sets that lead to a certain outcome. The approach is illustrated in Fig. 1 and consists of five steps:

### 1. Defining system response classes

**Figure 2:**
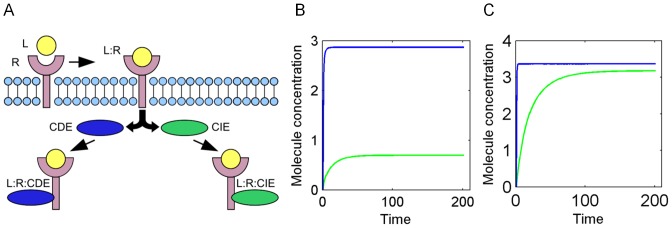
Pathways of EGF receptor internalization. EGF receptor can be internalized via clathrin dependent endocytosis (CDE) and clathrin independent endocytosis (CIE). **A**) Schematic representation of EGF receptor internalization according to Schmidt-Glenewinkel et al. [21]. **B**) Example trajectory representing class 0 (‘pathway off’) for clathrin dependent EGFR-internalization. **C**) Example trajectory representing class 1 (‘pathway on’) for clathrin independent EGFR- internalization.

We first define response classes for the model outcome. Since the method is applied to systems of signaling pathways, we are interested in a binary outcome represented by the classes 0 and 1 for ‘pathway inactive’ or ‘pathway active’, respectively. This is done by criteria that have been pre-defined for the trajectory of a key indicator of pathway activity. According to the shape of the species output trajectory of interest, formal criteria are defined to classify a system response as either class 0 or 1. To illustrate the definition of such classified system response, consider the first model of EGF-receptor internalization [21] (Fig. 2A). Here, the clathrin-independent endocytosis (CIE) pathway is defined as ‘activated’, if the level of activated receptor internalized via CIE reaches at least 80% of the level of the activated receptor internalized via clathrin dependent endocytosis (CDE). Note that those classification criteria can be arbitrarily defined with respect to the analyzed model and the specific scientific question.

### 2. Generation of matrix of random initial values

In order to sample the whole parameter space of initial concentrations for its ability to generate the system response defined in step 1, a matrix of random initial values drawn from a probability distribution within pre-defined ranges is generated. Throughout this study, we assume uniformly distributed parameters on an interval (Tables S1, S2 and S3 for models 1, 2, and 3, respectively)**.** Here, each column corresponds to a specific model species having a non-zero start value at simulation time t_0_. Each row vector relates to one parameter set of initial values used as an input to the ODE-system for one simulation run. Model species that require initial values are generally un-cleaved, un-complexed molecular species and typically represent proteins.

### 3. Model simulation and classification of generated parameter sets

Each parameter set of initial concentrations generated in step 2 is used as input to the model of ODEs. All numerical simulations were done using the ode15s function within MATLAB (The Mathworks, Natick, Massachusetts, United States). The MATLAB code for the three model simulations are given in Files S1-S3. The resulting output trajectories of interest are then classified according to the predefined criteria in step 1. Based on the result of classification, the parameter sets are assigned to class 0 or 1 (‘pathway active’ and ‘pathway inactive’, respectively).

### 4. Construction of a matrix of species relations

The assumption that relative rather than absolute molecule concentrations are crucial for pathway activation motivated us to construct a matrix containing categorized, pair-wise ratios of initial concentrations, based on the matrix of initial concentrations generated in step 2. For each parameter set, the relations of all unordered pairs of non-zero molecule species A and B are encoded as ‘0’ in case a_0_ < b_0_, as ‘1’ in case a_0_  =  b_0_ and as ‘2’ in case a_0_ > b_0_, with a_0_ (b_0_) being the initial concentration of A (B). Thus, the numerical values of initial values are transformed into categorical attributes of a parameter set. Since equal values for randomly generated numbers are highly unlikely, this procedure essentially comes down to binarization of concentration relations into categories 0 and 2, respectively.

### 5. Application of a decision tree algorithm to species relations

The matrix of species relations (step 4) together with the class information obtained from step 3 are then subjected as training data to a decision tree algorithm, which yields a tree representation or a rule set on molecule relations that lead to the system response as defined in step 1.

The resulting decision tree contains relations of the form a_0_ < b_0_, a_0_  =  b_0,_ or a_0_ > b_0_ as decision nodes, and class labels (1/0 for pathway active or pathway inactive) at leave nodes. The tree can further be translated into a rule set, where each path from the root to any leave node represents one rule to achieve a certain pathway activation status.

We use the MATLAB implementation of the CART (classification and regression trees) algorithm developed by Breiman et al. [7] to construct a binary classifier. In brief, the decision tree classifier is constructed by asking a sequence of hierarchical Boolean questions and thereby recursively partitioning the training data set. For this procedure every possible split over all features is considered and evaluated to choose the criterion for the best split. The goodness of split is evaluated by an impurity function, here the Gini index [7]. The aim of a good split is to partition the data into descendant subsets that are more homogeneous than the parent subset with regard to their assigned class. The default algorithm constructs the full decision tree first and then prunes it to yield subtrees that do not overfit the training data. The smallest subtree that is within one standard error of the minimum cost subtree is regarded as the tree pruned to the best level [7].

The performance of the tree was calculated by using a resubstitution method and a 10-fold cross validation [7] expressing the cost of the tree by means of the misclassification error on training and test data, respectively. We selected an equal number of sets from each class before applying the decision tree algorithm to the parameter sets to build an unbiased classifier.

## Results

### Decision tree based model analysis is able to classify system responses

We applied our method to a small model with a motif for competitive binding which has been adapted and formulated by Schmidt-Glenewinkel et al. [21] for investigation of the sorting mechanism of the EGF receptor into two different internalization pathways. Upon ligand activation, the receptor either internalizes via the clathrin-dependent pathway (CDE-pathway), or via the clathrin-independent pathway (CIE-pathway), resulting in the model species L:R:CIE and L:R:CDE, respectively, as the most downstream species (Fig. 2A). Schmidt-Glenewinkel et al. studied the conditions leading to a switch-like behavior of species L:R:CIE and hence activation of the CIE-pathway.

To derive conditions that are able to distinguish between the classes of activation (CIE-pathway on and off, respectively), we defined the system response based on the time courses of the model species L:R:CIE and L:R:CDE (step 1 of method). Output trajectories were considered as representatives for CIE-pathway activation, if the L:R:CIE level reaches 80% of the L:R:CDE level. Otherwise, trajectories were defined to represent pathway inactivation. Fig. 2B and Fig. 2C illustrate example trajectories for both classes.

Random initial values were generated for the initial concentration of the ligand, the receptor and the two adaptors CIE and CDE (Table S1) (step 2 of method) and used as input for model simulation. According to our predefined criterion in step 1, parameter sets were assigned to class 0 or 1 (‘pathway active’ and ‘pathway inactive’, respectively) (step 3 of our method). Out of 10,000 sampled parameter sets, we selected n  =  1808 sets in total (904 representatives for each class) as input for the decision tree learning algorithm.

**Figure 3:**
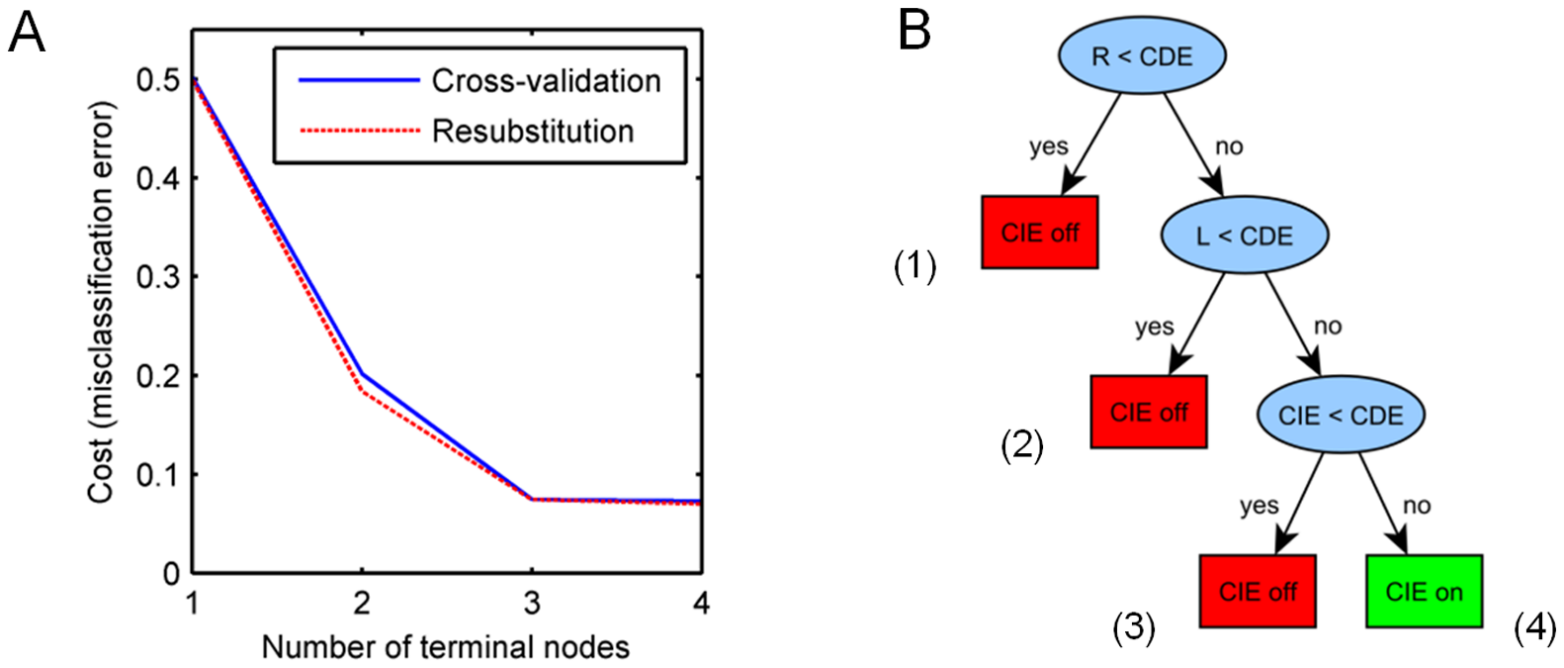
Misclassification error and decision tree for EGF receptor internalization. **A**) Misclassification error decreases depending on the number of terminal nodes of the decision tree in B). **B**) The full decision tree contains four terminal nodes and yields a misclassification of less than 1% for both training and test.

**Figure 4:**
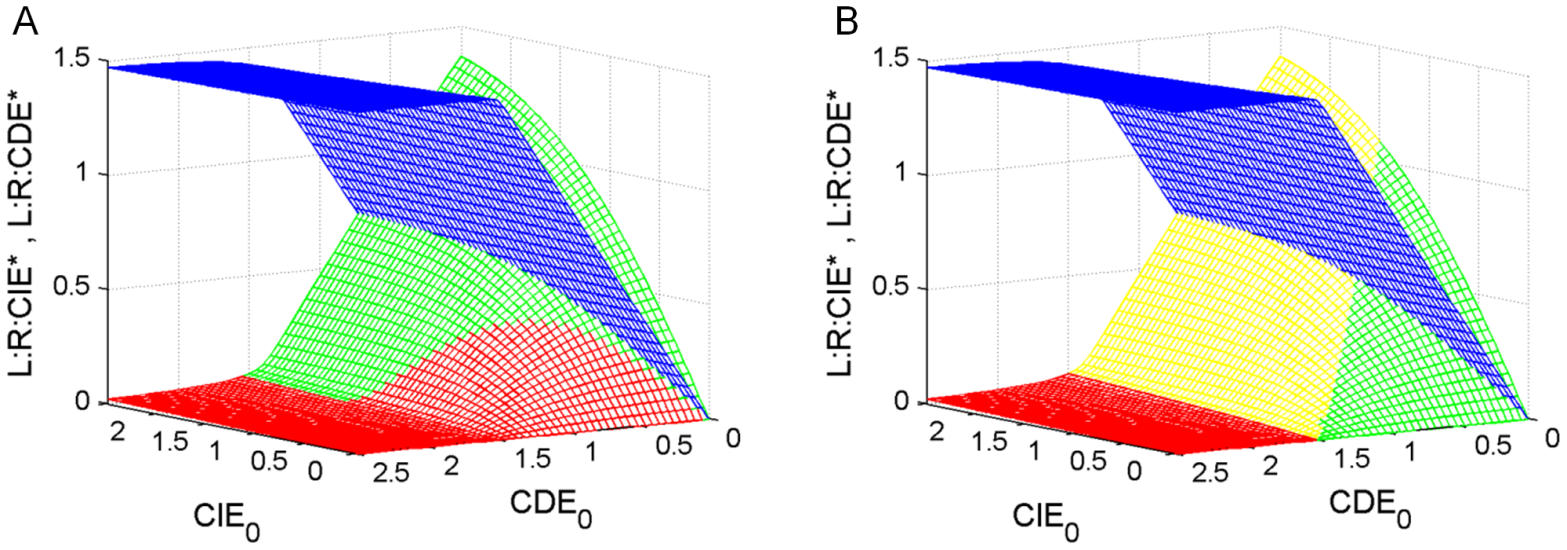
Comparision of conditions predicted by decision tree and previous findings in [21]. Dependence of internalized receptor levels on CIE_0_ and CDE_0_ adaptors after reaching steady state at t  =  200 (ligand and receptor initial values set to L_0_  =  R_0_  =  1.5. Receptors internalized via CDE are colored in blue, all other colors refer to receptors internalized via CIE. (cf. [21]; CIE_0_ and CDE_0_ 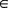 [0, 2.5]). The conditions for CIE-pathway activation derived from decision tree rules (green, panel A) show a large overlap with previously identified conditions by steady-state analysis and parameter scans (Table 2, (C2) and (C3)) [21], depicted in panel B in yellow and green. **A**) Receptors internalized via CIE for parameters fulfilling pathway activating conditions according to decision tree (rule D4 of Table 2) are coloured in green, all other conditions not fulfilling that rule are coloured in red. **B**) Steady state analysis of [21] for full pathway activation in green (Table 1, condition C3), for moderate activation in yellow (Table 1, condition C2) and no activation in red (Table 1, condition C1).

The misclassification error obtained from the algorithm (Fig. 3) decreases with regard to the number of leaf nodes for both training and test by cross-validation suggesting a decision tree containing four leaf nodes (Fig. 4) as the tree on the best level (see Methods section) with a misclassification error of less than 0.1 (Fig. 3). While the misclassification rate is decreasing with increasing complexity of the tree, the steepness of this curve as well as the absolute value of the error demonstrate that pathway activation can be sufficiently explained by already fairly simple rule sets.

### Decision tree based model analysis reproduces results obtained by steady state analysis and parameter scans

The focus of the earlier work by Schmidt-Glenewinkel et al. [21] was to elucidate conditions that lead to a switching behaviour of the CIE-pathway. This analysis was done by steady-state analysis and parameter scans. Two different steady-state classes were identified which are reached depending on the initial values of molecule species leading to either a low, medium or high number of ligand-activated receptors internalized via CIE-adaptors (Table 1).

**Table 1:** Conditions C1-C3 on initial values of species required for reaching a distinct steady-state class and activation level of clathrin-independent EGFR-internalization according to Schmidt-Glenewinkel et al. [21].

Initial values	Steady-state	CIE-internalization
(C1) min{L_0_,R_0_} < CDE_0_	I	Low
(C2) min{L_0_,R_0_} > CDE_0_	II	Medium
(C3) min{L_0_,R_0_} > CDE_0_ + CIE_0_	III	High

_0_ denotes the initial concentration of species X. X

The system reaches steady-state class I if all free receptors or all ligand molecules get depleted and all activated receptors (model species EGF:R) are internalized [21]. This steady state was associated with no or basal receptor internalization via CIE-adaptors and is reached in case the initial values of either ligand or receptor is lower than the number of CDE-adaptors (condition C1, Table 1).

For reaching steady state class II, neither the ligand nor the receptor is limiting with respect to pathway activation, but both adaptor types are consumed completely. This steady state is reached, if ligand and receptor concentrations exceed initial values of CDE adaptors leading to a moderate CIE-pathway activation (condition C2, Table 1). A high level of CIE-receptor internalization is reached if ligand and receptor concentrations exceed the sum of CIE_0_ and CDE_0_ (condition C3, Table 1).

To demonstrate how the conditions found by our method and stated in the decision nodes reflect the requirements of Schmidt-Glenewinkel et al. [21], we transformed the decision tree (Fig. 4) into a set of rules where each path from the root to any leaf node is represented as a rule containing the expressions of each decision node on that path (Table 2).

**Table 2:** Full rule set extracted from the decision tree of Fig. 4.

Path	Rule
(D1)	If R_0_ < CDE_0_ then class "CIE off"
(D2)	If R_0_ > = CDE_0_ and L_0_ < CIE_0_ then class "CIE off"
(D3)	If R_0_ > = CDE_0_ and L_0_ > = CDE_0_ and CIE_0_ < CDE_0_ then class "CIE off"
(D4)	If R_0_ > = CDE_0_ and L_0_ > = CDE_0_ and CIE_0_ > CDE_0_ then class "CIE on"

Fig. 4. Rules D1-D3 represent conditions for the systems response “no pathway activation”. Rule D4 suggests conditions for pathway activation. Rules D1-D4 correspond to paths (1)-(4) of the decision tree in

The top two decision nodes “R_0_ < CDE_0_” and ”EGF_0_ < CDE_0_” reflected in rule D1 and in the second expression of rule D2 of Table 2 indicate that either the ligand or the receptor level is required to be lower than CDE start concentration to result in an inactive CIE-pathway. This directly corresponds to condition C1 (Table 1). Our method thus identifies either the ligand or the receptor as the limiting factor for pathway activation, which is in complete agreement with [21] (Table 1).

For a moderate activation of the CIE-pathway, both the ligand and receptor initial values have to exceed the number of CDE-adaptors (condition C2, Table 1), which is reflected in the decision tree in the top two decision nodes combined with each other (first two terms of rule D4 for pathway activation (“R_0_ > =  CDE_0_” and “L_0_ > =  CDE_0_”, Table 2)).

**Figure 5:**
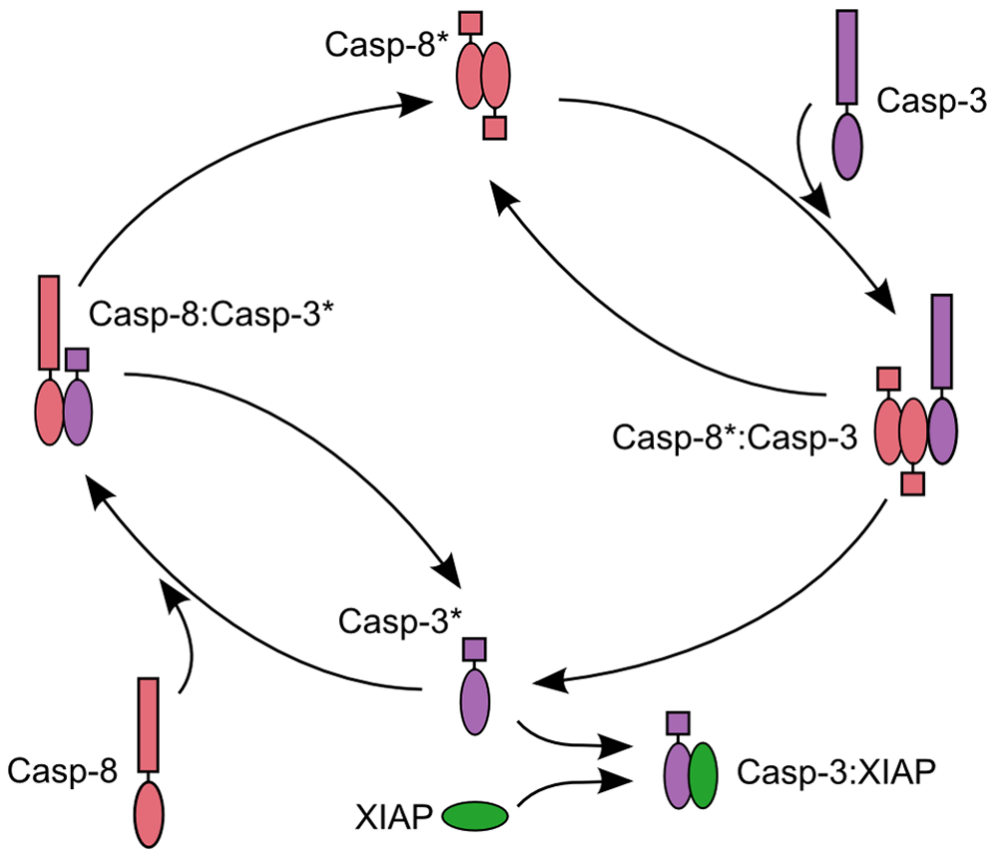
Molecule interaction scheme of the caspase-3 activation model in [3]. In brief, caspase-3 activity is regulated by caspase-8 and XIAP. Caspase-8 activates caspase-3 after forming a complex. Positive feedback is accomplished through activation of caspase-8 by caspase-3. XIAP inhibits active caspase-3. The asterisk denotes an active caspase.

However, for a full activation (condition C3, Table 1) leading the system towards steady state class II, ligand and receptor have to exceed the sum of CDE- and CIE-adaptors according to [21]. This condition cannot be directly extracted from the decision tree and its corresponding rule set. We therefore explored how initial values of pathway species influence pathway activation by simulation of receptors internalized via CIE and CDE at steady state (Fig. 5) [21]. Simulation results for L:R:CIE- and L:R:CDE- levels at steady state are illustrated according to the decision tree results in Fig. 4A and according to the conditions obtained by steady state analysis in combination with parameter scan (Table 1, [21]) in Fig. 4B.

The steady state levels of receptors internalized via CIE and classified as ‘pathway on’ (moderate or full) depicted in Fig. 4B in yellow and green show a large overlap with our decision tree result of Fig. 4A (‘pathway on’ coloured in green). However, the decision tree performs a binary classification (‘pathway on’ or ‘pathway off’ depicted in green and red respectively (Fig. 4A) instead of a three-fold categorization (full, moderate and off).

Further, our method suggests pathway activation in case initial CIE adaptor values exceed CDE adaptor values (CIE_0_ > CDE_0_), given that CDE_0_ < 1.5 ( =  L_0_, R_0_), i.e. given that there are potentially more ligand-activated receptors than CDE-adaptors. The condition CDE_0_ < min{L_0_, R_0_} was suggested by both approaches (see Table 1 (D2), (D3) for decision tree and Table 2 (C2) (C3) for analytical solving combined with parameter scan) and reflects the following context: The model in [21] contains rate constants for adaptor binding, where binding affinity of CDE-adaptors is higher than CIE-adaptors. Hence, the CDE-pathway gets activated faster. This leads to a fast depletion of ligand-activated receptor complexes (L:R) by binding to CDE-adaptors. In case min{L, R} < CDE_0_, all CDE-adaptors get depleted first, before remaining ligand-receptor complexes can associate with CIE-adaptors. Therefore a full activation in [21] corresponds to a steady state class, where both adaptor types get depleted, independent of the level of pathway activation (yellow in Fig. 4B). In case only CDE-adaptors get depleted, a moderate activation takes place according to [21] (yellow in Fig. 4B).

However, our method points out the effect of CDE-pathway saturation prior to CIE-pathway activation due to competitive binding, by identifying the condition CIE_0_>CDE_0_ for CIE-pathway activation and assuming threshold mechanisms as part of the method in first place.

Hence the decision tree categorizes the subspace for CIE_0_+CDE_0_ < L_0_ ( =  R_0_  =  1.5) as not activated (Fig. 4A). This is different from the analytical solution (Table 1), which identifies this subspace as ‘pathway activated’, however, at fairly low absolute values of L:R:CIE as well as low relative levels compared to L:R:CDE (Fig. 4B).

We further compared the decision tree result to pathway activation according to the predefined criterion for classification of parameter sets (step 1 of the methods section).

Here, the decision tree assigns a larger area to ‘pathway on’ (Fig. S1 A, green area) compared to the predefined criterion (Fig. S1 B, green area). This is due to simple rules of our approach used as features (A_0_ < B_0_). Parameter combinations that yield a L:R:CIE-level greater than 80% of the L:R:CDE-level (colored in green in Fig. S1) cannot be explained more precisely by our rules.

This results in the assignment of a larger area to ‘pathway on’ (Fig. S1 A, green area) compared to the predefined criterion (Fig. S1 B, green area). Nevertheless, the results show a large agreement for activation status between predefined conditions and predicted conditions for pathway activation.

Taken together, the here described method is able to accurately reproduce the previously published conditions leading to an inactive CIE-pathway. Further, conditions derived for an active CIE-pathway are largely in agreement with conditions described in [21].

### Decision tree and direct Lyapunov exponent analysis yield comparable results

**Figure 6:**
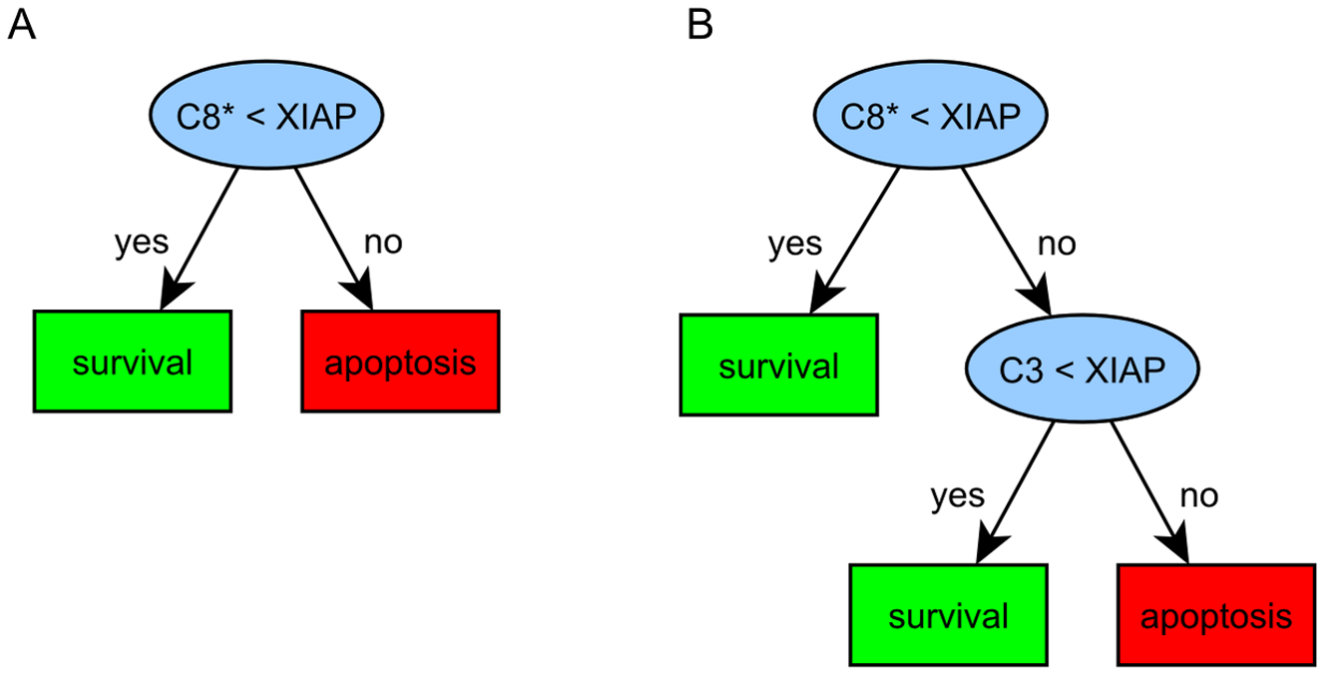
Decision trees resulting from analysis of the caspase activation model. Decision tree resulting from analysis of the model illustrated in Fig. 5 for a misclassification error of 25.7% (A) and 17.43% (B), respectively.

Next, we compared the performance of our decision tree method to an alternative for multivariate model analysis approach applied to signaling networks. Direct Lyapunov exponent (DLE) analysis is a multivariate method that identifies phase-space domains of high sensitivity to initial conditions. By applying DLE analysis to a core sub-network of caspase-3 activation, Aldridge et al. [3] identified regions in phase space, i.e., sets of initial species concentrations, that give rise to different system responses, here survival and programmed cell death (apoptosis), respectively [3]. Key factor for induction of apoptosis is a sufficiently high level of concentration for active caspase-3 (see below). Caspase-3 activity is regulated by a number of molecules (Fig. 6). The caspase-3 activation model in [3] contains eight molecular species including pro-apoptotic procaspase-3, caspase-3, procaspase-8 and caspase-8 and pro-survival factor XIAP as well as three intermediate products (Fig. 5).

We applied our decision tree method to this caspase-3 activation model by setting rate constants to the values in [3] and by using similar ranges for initial conditions of species concentration as in [3] (Table S2). Initial values for intermediate or processed species (i.e., active caspase-3 and any active caspase bound to a pro-caspase or XIAP) are assumed to be zero. Since the presence of active caspase-8 is required to initiate the cascade of caspase activation, the initial value for caspase-8 was set to the same range as in [3]. We then generated random initial concentrations for all four species (procaspase-8, active caspase-8, procaspase-3 and XIAP).

In ref. [3] cells were categorized as apoptotic if they exhibit a relatively tall and wide pulse of active caspase-3 [3] (for examples of caspase-3 trajectories representing apoptosis and survival see Fig. S2). Accordingly, we set the definition for classification of parameter sets to the class ‘apoptosis’ (class 1), if their resulting caspase-3 trajectories exhibit a pulse lasting for at least three hours at a level higher or equal to 1.5×10^5^ molecules per cell and to ‘survival’ (class 0) otherwise. The misclassification errors of all sub-trees obtained by the decision tree algorithm suggest a tree consisting of six leaf nodes (Fig. S4) as the tree on the best pruning level with a misclassification error of 0.14. Note that even decision trees with two or three leaf nodes show good performance as indicated by a considerably low misclassification error (26% and 18%, respectively, see Fig. S3).

The top decision node suggested by our analysis identifies the relation of active caspase-8 and XIAP as the most important feature (Fig. 6A), which accounts for the reduction of the misclassification error to 25.7%. This is very much in accordance with [3] that identified the balance between active caspase-8 and XIAP to influence cell fate: the higher the concentration of active caspase-8, the higher XIAP levels are needed to cross the separatrix of large DLE values from death to survival [3]. The same notion is reflected here by the top decision node containing the relation of active caspase-8 and XIAP (Fig. 6A) where a XIAP level exceeding that of active caspase-8 is linked to survival, while caspase-8 concentration higher than XIAP is associated with apoptosis.

The leaf node representing the class “survival” in Fig. 6A yields class probability of 1.0 and therefore corresponds to a pure subset due to the split of the top decision node. However, caspase-8 exceeding XIAP can still lead to survival in cases where procaspase-3 concentration is lower than XIAP, as indicated by addition of the second decision node (Fig. 6B) leaving 17.43% parameter sets as falsely classified to cause apoptosis.

Despite a clear reduction of misclassification errors with increasing numbers of nodes, the strongest reduction of misclassification is achieved by the first split induced by the top node. This simple tree is able to correctly classify 74.3% of the generated parameter sets. Note that in comparison to the DLE based analysis in [3], our method does not require visualization and exploration of phase space and yet comes to the same conclusions by directly suggesting simple rules from a readily constructed decision tree. Further, our approach requires only 50.000 parameter sets to identify comparable conditions on pathway activation instead of 1.594.323 different parameter sets that were used by Aldridge et al. Hence our approach is computationally less expensive than the DLE based analysis.

### Analysis of a comprehensive apoptosis model identifies known antagonists of apoptosis signaling

**Figure 7:**
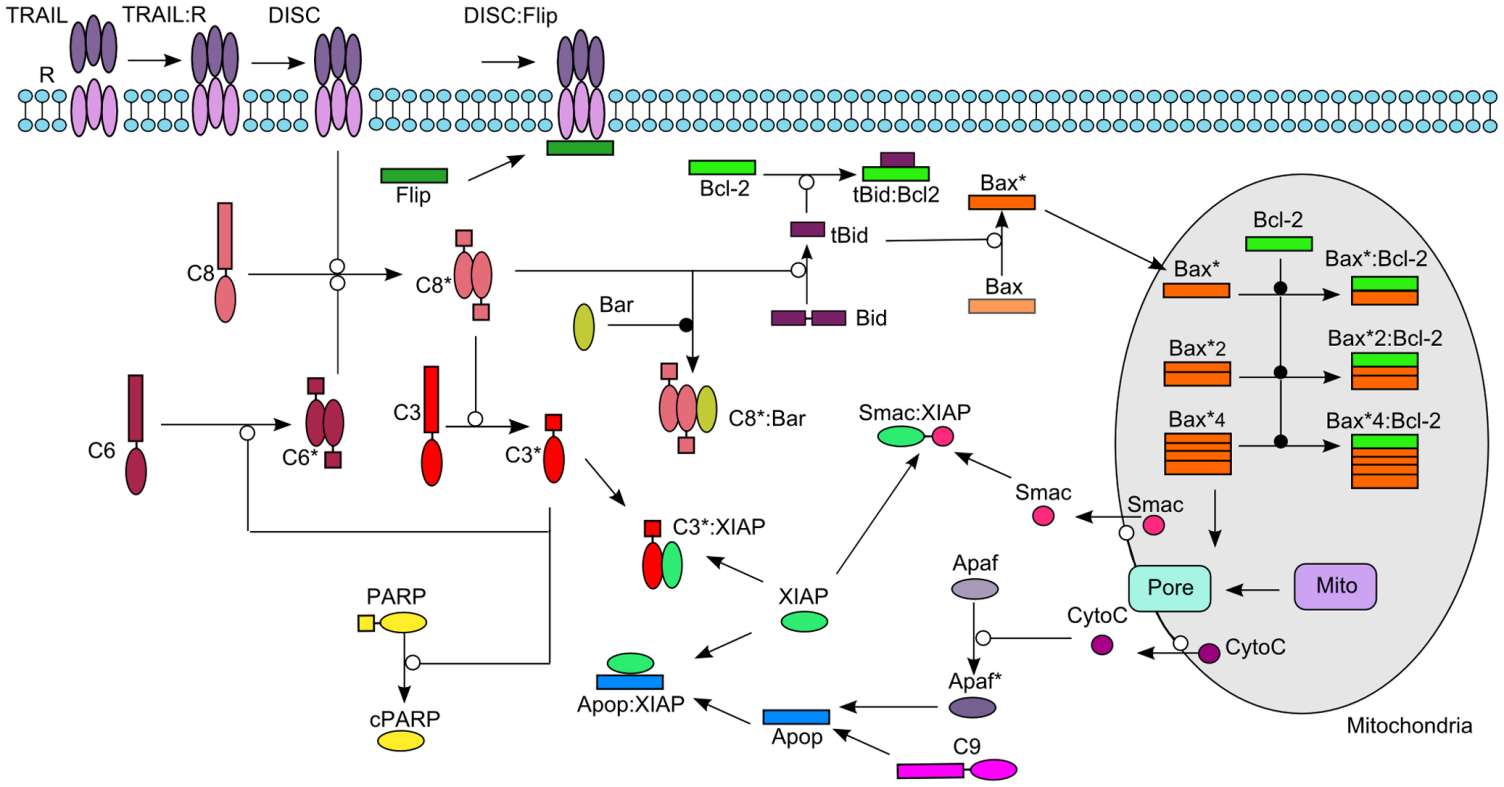
Model scheme of TRAIL induced apoptosis. Process diagram of the TRAIL induced apoptosis model by Albeck et al. [2]. Active species are denoted by an asterisk.

Programmed cell death (apoptosis) is an inherently complex signaling process that is regulated by a considerably large numbers of molecules. When exposed to tumor necrosis factor (TNF) or TNF-related apoptosis-inducing ligand (TRAIL) only upstream initiator caspases are active for a variable delay time. A subsequent and sudden transition leads to activation of the downstream effector caspases that induce rapid cell death. Albeck et al. [2] developed a mathematical reaction model based on mass-action kinetics of TRAIL induced apoptosis. This model (EARM v1.0) contains 58 equations of which 18 species have non-zero initial values (Fig. 7). Based on their model analysis, they concluded that the duration of the delay prior to effector caspase activation is determined by initiator caspase-8 activity and the rates of other reactions immediately downstream of the TRAIL receptor. Sudden, subsequent activation of effector caspases is achieved by reactions involved in permeabilization of the mitochondrial membrane and relocalization of proteins such as Smac.

Here, we categorize the system response into two distinct classes, “apoptosis” (class 1) and “survival” (class 0) and use the amount of cleaved PARP as an indicator for effector caspase activity. We then defined the class of apoptosis when the delay time T_s_ for activating the effector caspases was less than 30 minutes and if at least 50% of PARP is cleaved.

**Figure 8:**
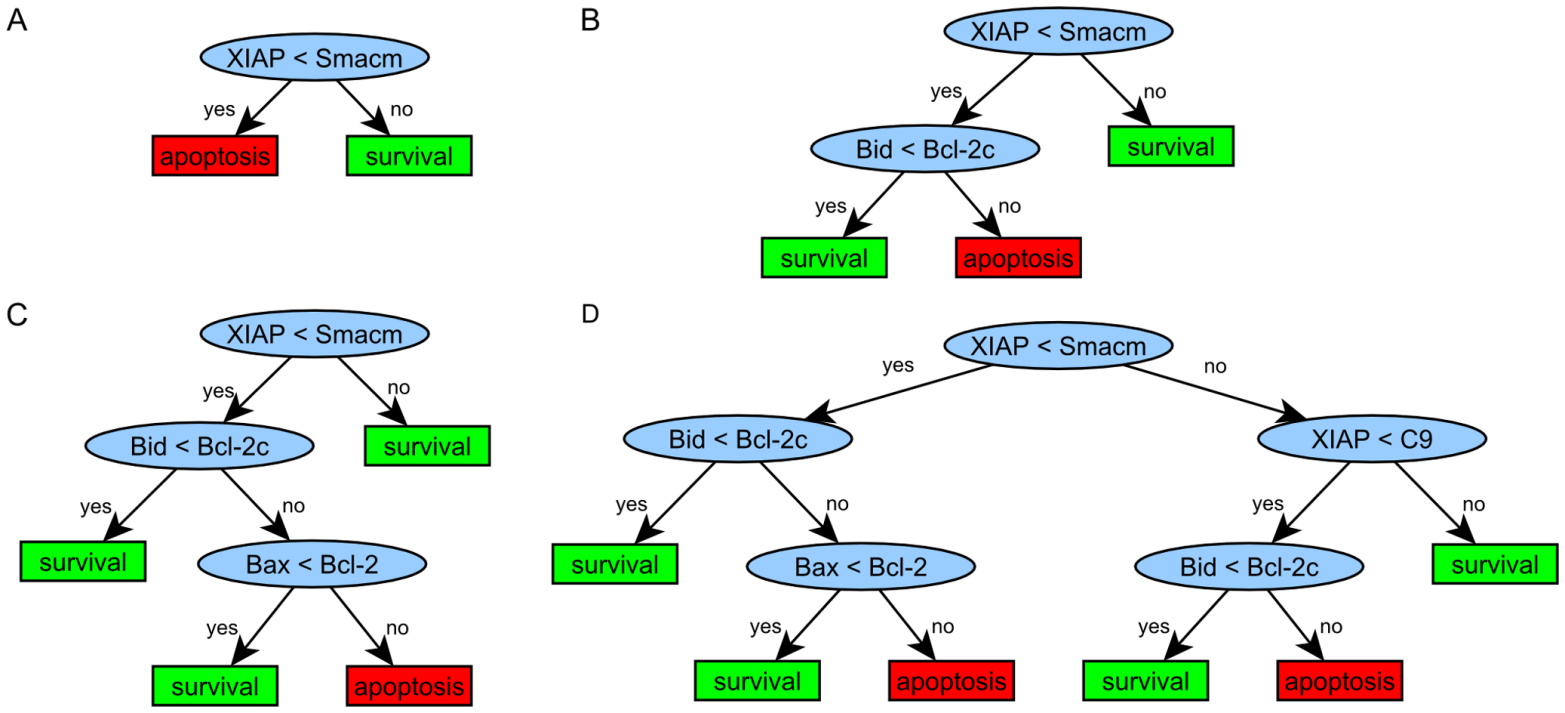
Decision trees resulting from analysis of apoptosis model EARM v1.0. Analysis of apoptosis model EARM v1.0 (Albeck et al. [2], illustrated in Fig. 7) results in decision trees (A) (B) (C) and (D) for a misclassification error of 30.85% (A), 24.68% (B), 21.17% (C), and 18.13% (D), respectively.

We then generated 100,000 random parameter sets within a range of four orders of magnitude centred at initial values reported in [2] for the afore-mentioned 18 molecular species. We chose 93,046 parameter sets (46,523 per class) as input for the decision tree algorithm. We obtained a rapid decrease in misclassification error with increasing number of leaf nodes in particular for trees with less than six leaf nodes (Fig. S5). With only one decision node a misclassification rate of approx. 30% is achieved, whereas five decision nodes decrease the misclassification error to approx. 18% (Fig. 8).

The concentration of XIAP compared to the one of Smacm representing mitochondrial Smac (second mitochondria-derived activator of caspases) is the highest ranked rule by our decision tree-based model analysis suggesting survival in case of mitochondrial Smac concentrations exceeding XIAP and apoptosis otherwise (Fig. 8A) [1], [2].

Smac/DIABLO (Direct IAP Binding Protein With Low pI) is a protein released from the mitochondria, which physically interacts with IAPs relieving the inhibitory effect of IAPs on caspase-3 and thereby sensitizing cells for apoptosis [8], [23]. It was shown by Verhagen et al. [23] that the ratio of IAP to DIABLO determines whether cells undergo apoptosis or not [2]. This experimental finding is in line with this top ranked decision rule.

The second ranked decision rule suggests that higher Bid concentrations compared to cytosolic Bcl-2 concentrations (Bcl-2c) lead to cell death given that mitochondrial Smac exceeds XIAP (top ranked rule). These two decision nodes account for a misclassification rate of 24.68% adding a second rule to the decision tree describing the relationship between known apoptosis antagonists [24] (Fig. 8B). The third-level node containing Bax and mitochondrial Bcl-2 in combination with the root node and the second decision node accounts for 21.17% of misclassification rate. As for the first and the second node, the third decision node suggests relations of molecular species that have been integrated into the model as direct interaction partners and that represent antagonists for cell fate decision making between apoptosis and survival [19].

When further increasing the number of leaf nodes in the decision tree, an alternative path from root to bottom assuming XIAP levels higher than mitochondrial Smac concentrations is obtained adding two decision nodes within one step (Fig. 8D). For concentrations of XIAP exceeding both mitochondrial Smac and procaspase-9 cell survival is predicted. Concentrations of XIAP lower than procaspase-9 and Bid concentration higher than cytosolic Bcl-2 levels lead to apoptosis whereas molecule concentrations of Bid lower than cytosolic Bcl-2 together with the upper level rules are associated with survival. The resulting tree has a misclassification rate of 18.13%.

Compared to the known antagonists Bid and Bcl-2, the importance of the apoptosis inhibitor XIAP compared to pro-apoptotic caspase-9 is less obvious, since these molecules are not direct interaction partners in EARM v1.0. In the model, caspase-9 is involved in formation of the apoptosome followed by caspase-3 activation, and XIAP is modeled as a direct inhibitor of caspase-3. Hence, the decision node describing the relation of XIAP and caspase-9 reflects the balance between caspase-3 activation and caspase-3 inhibition to be an important relation deciding on cell death or survival. For XIAP exceeding both Smac and procaspase-9, our decision tree suggests that Bid concentrations exceeding cytosolic Bcl-2 can overcome XIAP inhibition and lead to apoptosis by Bid sequestering cytosolic Bcl2 [13]. In case active caspase-8 is present, Bid can be cleaved to tBid and further enhance the activity of the pro-apoptotic pathway [2], [4], [5], [9], [11], [15].

In summary, the here presented decision tree based method for model analysis is capable to identify relationships between molecular species that are in accordance with experimental findings in earlier studies. Compared to alternative methods for multivariate sensitivity analysis our approach is able to produce comparable results with a conceptually simpler and computationally less expensive approach. The final case study exemplifies that the here described approach for model analysis can be readily applied even to complex signaling models making our method an important tool for model analysis in various fields of systems biology.

## Discussion

We developed a novel method for analysis of mathematical models of signaling networks. Given an ODE-based model of a signaling pathway we perform model simulations with randomly generated initial values for parameters that are then transferred into a decision tree algorithm with the aim to generate hierarchical rule sets that can be related to different predefined pathway states. As expected for a decision tree based classification, the misclassification error decreases with increasing number of leaf nodes. In the three model examples studied in this paper a high accuracy classification accuracy of pathway states is already obtained for only a small number of nodes suggesting that only few molecular species and the relationships among them control the system response.

The idea of using regression trees to evaluate and select pairwise associations of variables (rules) was previously described by Ishwaran [13]. However, the interpretation of these rules with respect to interactions among associated variables remained a challenge. Here, we approached this problem by first using relations between parameters instead of absolute parameter values as input to the decision tree. Second, we took advantage of the frequently observed switch-like behaviour of signaling pathways that switch between system states depending on changes in molecular concentrations. These different, pre-defined system states were then used as classification classes within the decision tree analysis.[6]

The method presented here relies on pre-defined rules for the classification of output trajectories. We did this mainly to be able to compare our results with those obtained by the authors of the models that have been presented. It is not an absolute requirement, though. It is possible to automatically detect classes by unsupervised learning. To demonstrate this, we have used k-means clustering on 50,000 trajectories that were simulated with the model from Aldridge et al. [3] from random parameter sets (Fig. S6, Text S1). Stability analysis of the clusters suggested that a two-class model was most appropriate (Text S1). Although the numbers of trajectories classified into either class was different between the supervised approach mentioned above and the unsupervised class discovery (Text S1), the centroids of the two classes were virtually indistinguishable (Fig. S6). This suggests that the difference of the number of trajectories classified as "apoptosis" can be explained by those that are in between both classes, or rather close to the centroid of "apoptosis", but just fall on the other side of the threshold that is introduced by formulating rules from prior knowledge. We thus conclude that the method can be easily extended to employ unsupervised clustering of output trajectories to define classes; the decision tree approach can be used equally well on those to derive rules on how the output changes with relations of parameters.

It is possible to introduce further constraints in the choice of the random drawings from the space of initial concentrations. This may be the case if additional knowledge suggests that combinations of concentrations of certain compunds are biologically implausible while the concentrations themselves are still in concordance with the initially defined ranges.

The decision tree based on parameter relations results in simple rules readily obtained from the tree. This method does not require visualization of the phase space like in DLE analysis, another multivariate method for model analysis that has been used here in comparison to our method. The simple rules and intuitively understandable presentation of hierarchical control strategies might come at the price of potential over-simplification. A limitation of our approach is that we cannot identify complex conditions, e.g., non-linear parameter relations. Further, since we only operate on parameter relationships we cannot determine quantitatively how absolute changes in molecular abundances are related to changes in system states.

These limitations can be overcome by using sub-symbolic alternative classification schemes such as artificial neural networks or support vector machines that are able to capture non-linear relationships between parameters. Further, we could use ratios of sampled parameters instead of discretized relationships between parameters to link absolute molecular abundances to system state changes. In both cases, however, the intuitive representation and understanding of conditions linked to specific system states would be lost. Despite this simplified model analysis framework we showed that in the three case studies for model analysis in the present paper we were able to identify all major parameter relationships that have been previously reported using alternative computational and experimental approaches, respectively [2], [4], [5], [9], [11], [15].

In summary, we present here a novel method for multivariate model analysis that is inherently simpler and more intuitive than alternative approaches and thus adds an important new methodology to the field of computational systems biology.

## Supporting Information

Figure S1 **Comparison of predefined classes and earlier reported conditions required for EGFR internalization.** Dependence of internalized receptor levels on CIE_0_ and CDE_0_ adaptors is illustrated after reaching steady state at t  =  200, (ligand and receptor initial values set to L_0_  =  R_0_  =  1.5). For comparison, the amount of receptors internalized via CDE is plotted in blue. **A)** Amount of receptors internalized via CIE for parameters fulfilling pathway activating conditions (rule D4 of Table 2) are coloured in green, all other conditions not fulfilling that rule are coloured in red. **B)** Amount of receptors internalized via CIE according to predefined classification criterion for pathway activation in green and red otherwise.
(TIF)

Figure S2 **Representative caspase-3 trajectories for apoptosis and survival according to** [**3**] **.** Cells were categorized as apoptotic by if they exhibit a relatively tall and wide pulse of active caspase-3 (blue curve) and as non-apoptotic in case of a low pulse (green curve) [3].
(TIF)

Figure S3 **Misclassification error depending on the number of leaf nodes for the caspase activation model.** Analysis of the caspase activation model (Fig. 5) results in a decreasing misclassification error for an increasing number of terminal nodes of the decision tree illustrated in Figs. 6 and S4.
(TIF)

Figure S4 **Full decision tree for the caspase-3 activation model.** Analysis of the caspase-3 activation model (Fig. 5) developed by Aldridge et al. [3] results in a decision tree containing six terminal nodes.
(TIF)

Figure S5 **Misclassification error depending on the number of leaf nodes for EARM v1.0.** Analysis of the apoptosis model EARM v1.0 results in a decreasing misclassification error for an increasing number of terminal nodes of the decision tree illustrated in Fig. 8.
(TIF)

Figure S6 **Centroids of model trajectories (activated caspase 3) from the caspase model of Aldridge et al.** [**3**] **obtained by simulation based on 50,000 random sets of parameters.** (A) Output classes have been determined by pre-defined rules, as described in the manuscript. Blue, survival; red. apoptosis. (B) Output classes have been determined by k-means clustering, as described in Text S1. Shown is the mean of the trajectories for either class (solid lines) +/- one standard deviation.
(TIF)

Table S1 **Parameters and ranges of them used for model simulation of model 1.**
(DOCX)

Table S2 **Parameters and ranges of them used for model simulation of model 2.**
(DOCX)

Table S3 **Parameters and ranges of them used for model simulation of model 3.**
(DOCX)

File S1 **MATLAB code for EGF internalization model.**
(M) Click here for additional data file.

File S2 **MATLAB code for apoptosis model from Aldridge et al.** [**3**] **.**
(M) Click here for additional data file.

File S3 **MATLAB code for apoptosis model from Albeck et al.** [**2**] **.**
(M) Click here for additional data file.

Text S1 **Additional methods and results for unsupervised clustering of trajectories obtained by simulation on the caspase-3 activation model from 50,000 random parameter sets.**
(DOCX)
