# Supplementary figures and images for: Decision-Tree Based Model Analysis for Efficient Identification of Parameter Relations Leading to Different Signaling States
Source: PLoS One. 2013 Dec 18;8(12):e82593. doi: 10.1371/journal.pone.0082593 (PMC3867358; doi:10.1371/journal.pone.0082593)

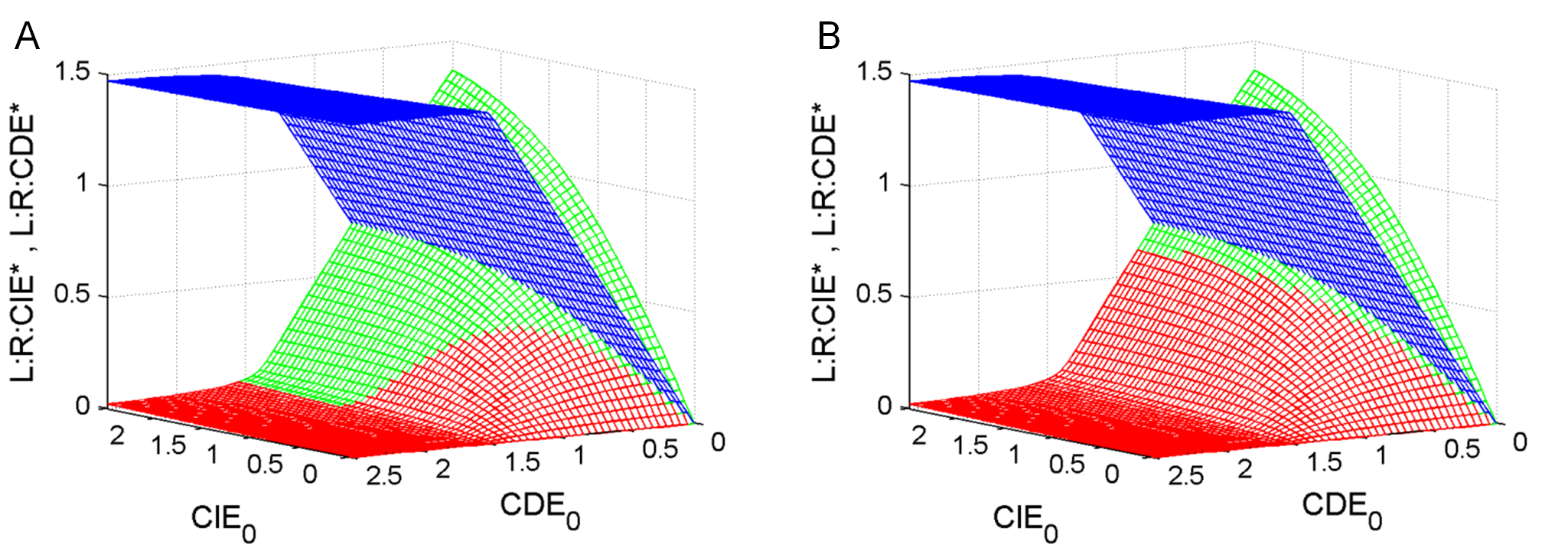

Supplement: Figure S1 — Comparison of predefined classes and earlier reported conditions required for EGFR internalization. Dependence of internalized receptor levels on CIE0 and CDE0 adaptors is illustrated after reaching steady state at t = 200, (ligand and receptor initial values set to L0 = R0 = 1.5). For comparison, the amount of receptors internalized via CDE is plotted in blue. A) Amount of receptors internalized via CIE for parameters fulfilling pathway activating conditions (rule D4 of Table 2) are coloured in green, all other conditions not fulfilling that rule are coloured in red. B) Amount of receptors internalized via CIE according to predefined classification criterion for pathway activation in green and red otherwise. (TIF) [file pone.0082593.s001.tif]

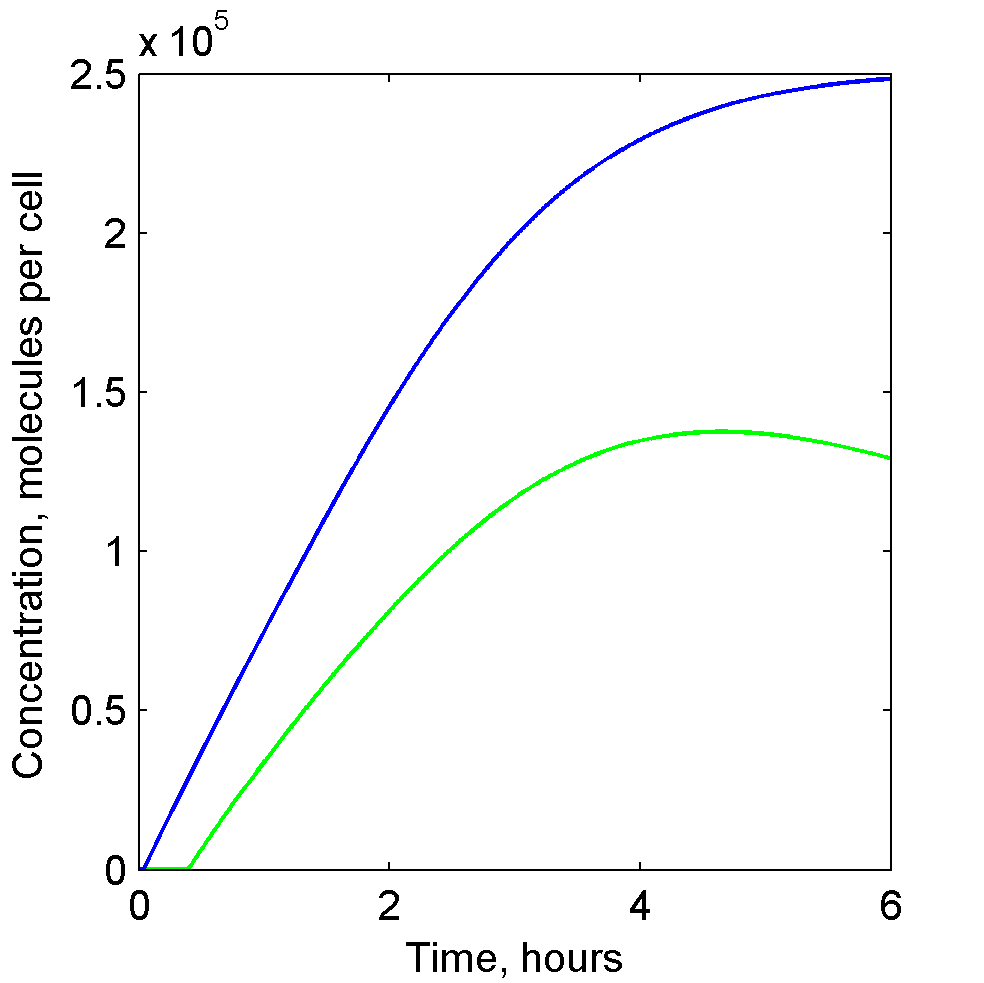

Supplement: Figure S2 — Representative caspase-3 trajectories for apoptosis and survival according to [3] . Cells were categorized as apoptotic by if they exhibit a relatively tall and wide pulse of active caspase-3 (blue curve) and as non-apoptotic in case of a low pulse (green curve) [3]. (TIF) [file pone.0082593.s002.tif]

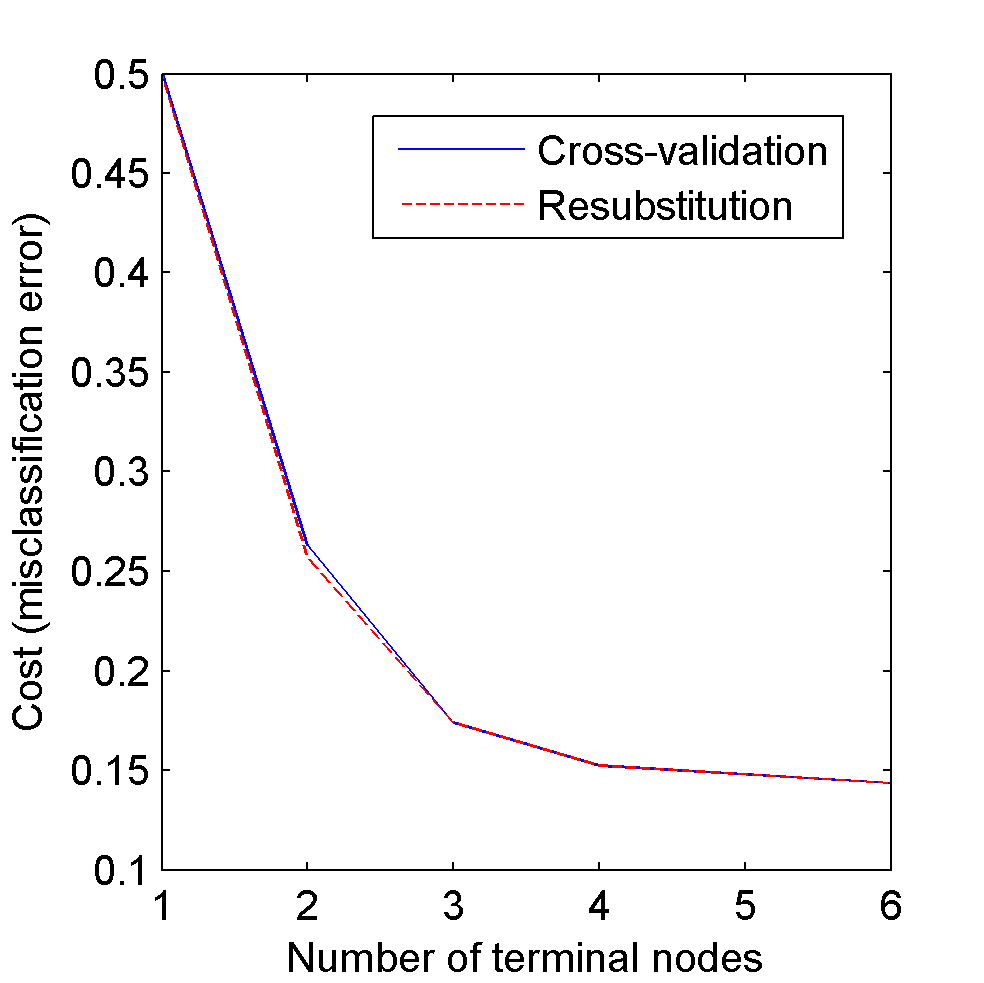

Supplement: Figure S3 — Misclassification error depending on the number of leaf nodes for the caspase activation model. Analysis of the caspase activation model (Fig. 5) results in a decreasing misclassification error for an increasing number of terminal nodes of the decision tree illustrated in Figs. 6 and S4. (TIF) [file pone.0082593.s003.tif]

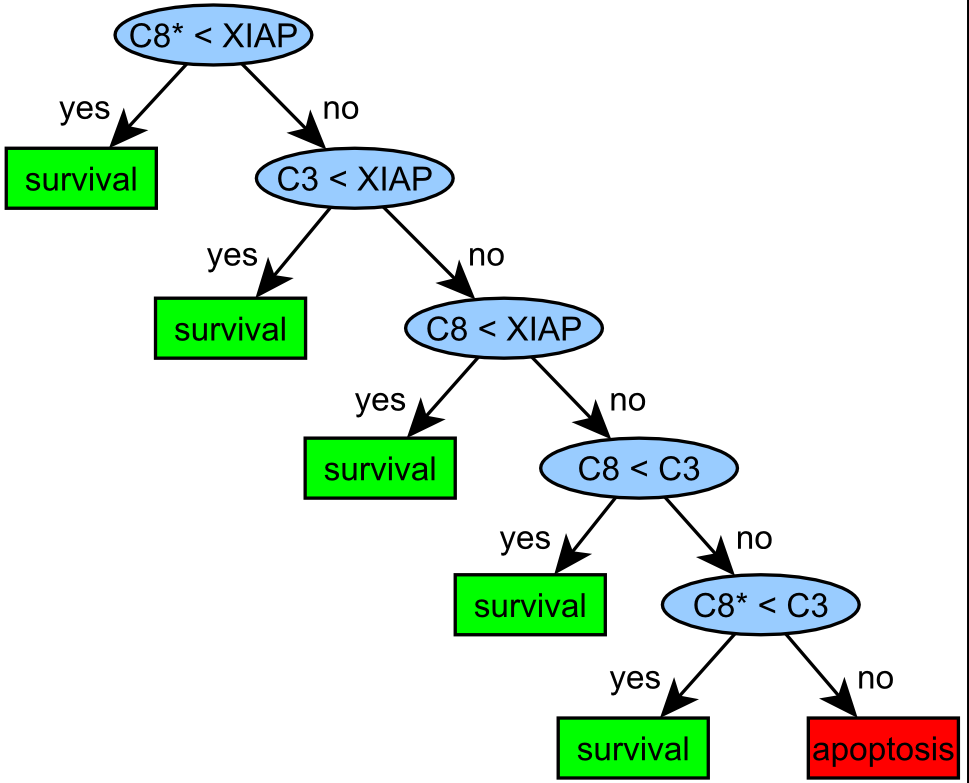

Supplement: Figure S4 — Full decision tree for the caspase-3 activation model. Analysis of the caspase-3 activation model (Fig. 5) developed by Aldridge et al. [3] results in a decision tree containing six terminal nodes. (TIF) [file pone.0082593.s004.tif]

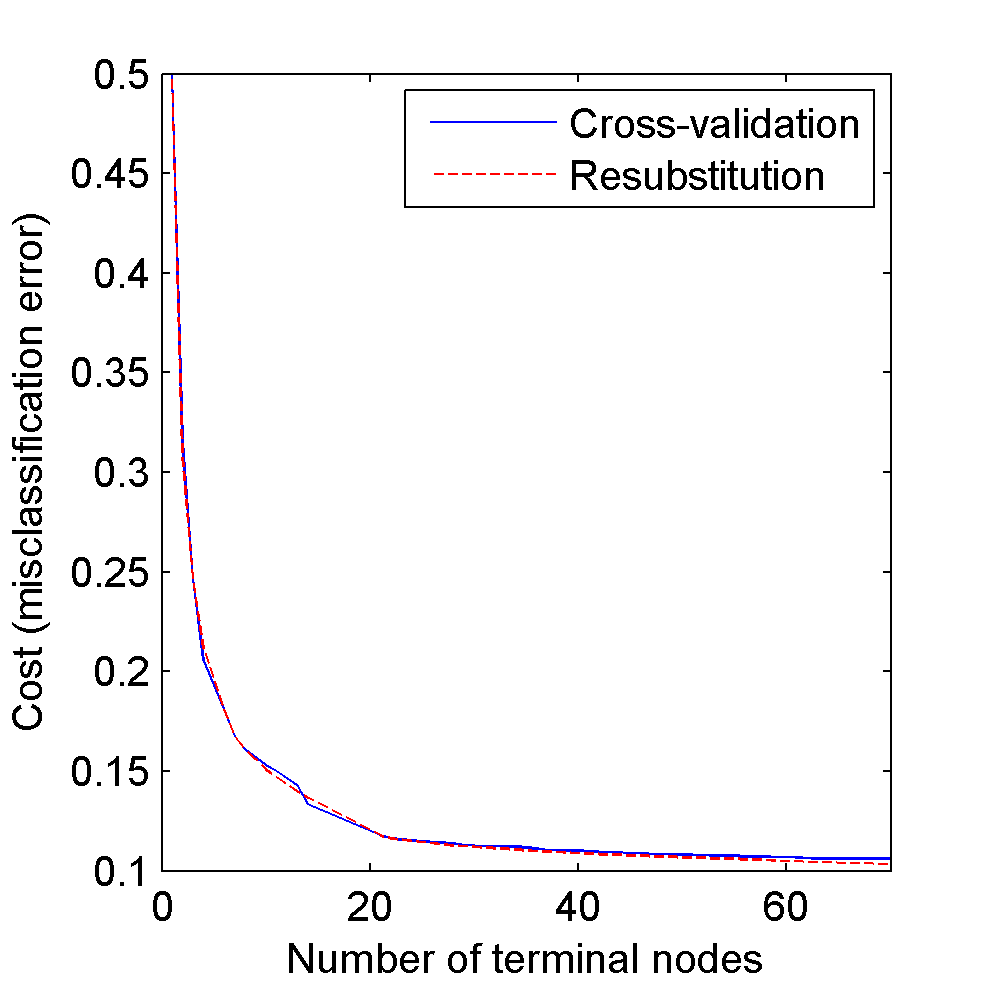

Supplement: Figure S5 — Misclassification error depending on the number of leaf nodes for EARM v1.0. Analysis of the apoptosis model EARM v1.0 results in a decreasing misclassification error for an increasing number of terminal nodes of the decision tree illustrated in Fig. 8. (TIF) [file pone.0082593.s005.tif]

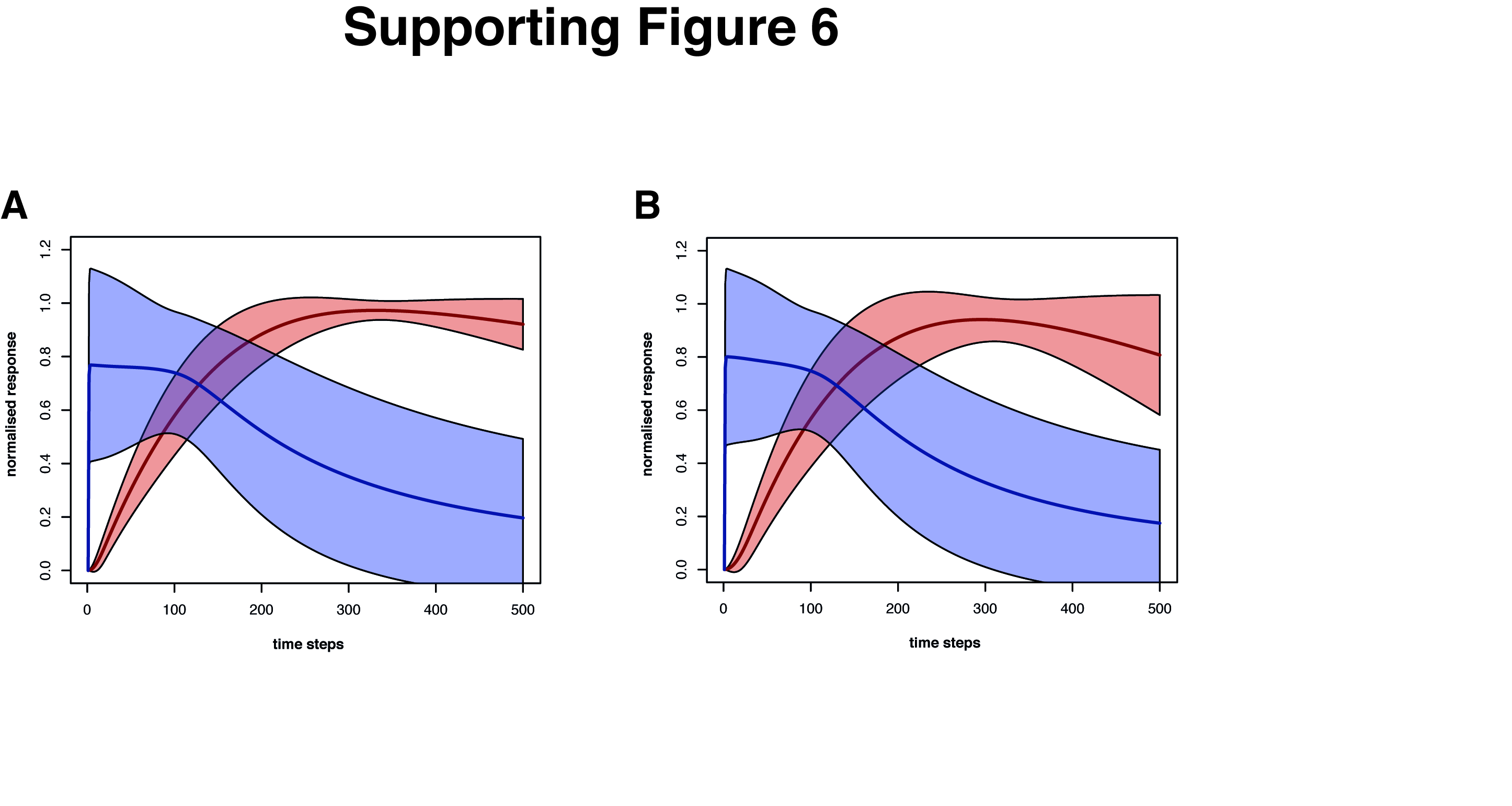

Supplement: Figure S6 — Centroids of model trajectories (activated caspase 3) from the caspase model of Aldridge et al. [3] obtained by simulation based on 50,000 random sets of parameters. (A) Output classes have been determined by pre-defined rules, as described in the manuscript. Blue, survival; red. apoptosis. (B) Output classes have been determined by k-means clustering, as described in Text S1. Shown is the mean of the trajectories for either class (solid lines) +/- one standard deviation. (TIF) [file pone.0082593.s006.tif]
