# Supplementary material for: Decision-Tree Based Model Analysis for Efficient Identification of Parameter Relations Leading to Different Signaling States
Source: PLoS One. 2013 Dec 18;8(12):e82593. doi: 10.1371/journal.pone.0082593 (PMC3867358; doi:10.1371/journal.pone.0082593)
Supplement: Table S1 — Parameters and ranges of them used for model simulation of model 1. (DOCX) [file pone.0082593.s007.docx]

**Supporting Information File 4**

**Supplemental Table S1: Parameters and their ranges for model 1**

| Variable | State | Description | Original IC | Range (Min) | Range (Max) |
| --- | --- | --- | --- | --- | --- |
| x_1_ | [EGF] | Ligand | 1.5 | 0.1 | 10 |
| x_2_ | [EGFR] | Receptor | 1.5 | 0.1 | 10 |
| x_3_ | [L:EGFR] | Ligand-Receptor Complex | 0 | 0 | 0 |
| x_4_ | [CIE] | CIE-adaptors | 2.5 | 0.1 | 10 |
| x_5_ | [L:R:CIE] | Ligand activated receptor internalized via CIE | 0 | 0 | 0 |
| x_6_ | [CDE] | CDE-adaptors | 2.5 | 0.1 | 10 |
| x_7_ | [L:R:CDE] | Ligand activated receptor internalized via CDE | 0 | 0 | 0 |
