# Supplementary material for: Decision-Tree Based Model Analysis for Efficient Identification of Parameter Relations Leading to Different Signaling States
Source: PLoS One. 2013 Dec 18;8(12):e82593. doi: 10.1371/journal.pone.0082593 (PMC3867358; doi:10.1371/journal.pone.0082593)
Supplement: Table S2 — Parameters and ranges of them used for model simulation of model 2. (DOCX) [file pone.0082593.s008.docx]

**Supporting Information File 5**

**Supplemental Table S2: Parameters and their ranges for model 2**

| Variable | Symbol | Description | Original IC (Min, Max) | Range (Min) | Range (Max) |
| --- | --- | --- | --- | --- | --- |
| x_1_ | [PC8] | Procaspase-8 | 100, 3.5e+05 | 0 | 3.5e+05 |
| x_2_ | [C8] | Active caspase-8 | 100, 1.0e+05 | 0 | 3.5e+05 |
| x_3_ | [PC3] | Procaspase-3 | 100, 3.5e+05 | 0 | 3.5e+05 |
| x_4_ | [C3] | Active caspase-3 | 100, 1.0 e+05 | 0 | 0 |
| x_5_ | [PC8:C3] | Procaspase-8 bound to active caspase-3 | 100, 2.5e+04 | 0 | 0 |
| x_6_ | [C8:PC3] | Active caspase-8 bound to procaspase-3 | 100, 2.5e+04 | 0 | 0 |
| x_7_ | [XIAP] | XIAP (X-linked inhibitor of apoptosis) | 100, 1.0e+05 | 0 | 3.5e+05 |
| x_8_ | [C3:XIAP] | Active caspase-3 bound to XIAP | 100, 2.5e+04 | 0 | 0 |
