# Supplementary material for: Decision-Tree Based Model Analysis for Efficient Identification of Parameter Relations Leading to Different Signaling States
Source: PLoS One. 2013 Dec 18;8(12):e82593. doi: 10.1371/journal.pone.0082593 (PMC3867358; doi:10.1371/journal.pone.0082593)
Supplement: Table S3 — Parameters and ranges of them used for model simulation of model 3. (DOCX) [file pone.0082593.s009.docx]

**Supporting Information File 6**

**Supplemental Table S6: Parameters and their ranges for model 3.**

| Variable | Symbol | Description | Original IC | Range (Min) | Range (Max) |
| --- | --- | --- | --- | --- | --- |
| x_1_ | [L] | death ligand | 3 × 10^3^ | 3 × 10 | 3 × 10^5^ |
| x_2_ | [R] | inactive receptor complex | 2 × 10^2^ | 2 | 2 × 10^4^ |
| x_3_ | [L:R] | ligand-receptor complex | 0 | 0 | 0 |
| x_4_ | [R*] | active receptor complex | 0 | 0 | 0 |
| x_5_ | [flip] | binds to active receptor acting as inhibitor | 1× 10^2^ | 1 | 1× 10^4^ |
| x_6_ | [flip:R*] | complex of flip and active receptor | 0 | 0 | 0 |
| x_7_ | [C8] | procaspase-8 | 2 × 10^4^ | 2 × 10^2^ | 2 × 10^6^ |
| x_8_ | [C8:R*] | procaspase-8 bound to active recpetor | 0 | 0 | 0 |
| x_9_ | [C8*] | procaspase-8 and procaspase-10 | 0 | 0 | 0 |
| x_10_ | [Bar] | binds to C8* and acts as an inhibitor | 1 × 10^3^ | 1 × 10^1^ | 1 × 10^5^ |
| x_11_ | [C8*:Bar] | complex of active caspase-8/10 and Bar | 0 | 0 | 0 |
| x_12_ | [C3] | procaspase-3 | 1 × 10^4^ | 1 × 10^2^ | 1 × 10^6^ |
| x_13_ | [C8*:C3] | complex of active caspase-8/10 and procaspase-3 | 0 | 0 | 0 |
| x_14_ | [C3*] | active caspase-3 | 0 | 0 | 0 |
| x_15_ | [C6] | procaspase-6 | 1 × 10^4^ | 1 × 10^2^ | 1 × 10^6^ |
| x_16_ | [C3*:C6] | complex of active caspase-3 and procaspase-6 | 0 | 0 | 0 |
| x_17_ | [C6*] | active caspase-6 | 0 | 0 | 0 |
| x_18_ | [C6*:C8] | complex of active caspase-6 and procaspase-8 | 0 | 0 | 0 |
| x_19_ | [XIAP] | X-linked inhibitor of apoptosis (XIAP) | 1 × 10^5^ | 1 × 10^3^ | 1 × 10^7^ |
| x_20_ | [XIAP:C3*] | complex of XIAP ans active caspase-3 | 0 | 0 | 0 |
| x_21_ | [PARP] | DNA damage repair enzyme, here represents all substrates of C3* | 1 × 10^6^ | 1 × 10^4^ | 1 × 10^8^ |
| x_22_ | [C3*:PARP] | complex of active caspase-3 and PARP | 0 | 0 | 0 |
| x_23_ | [cPARP] | cleaved PARP | 0 | 0 | 0 |
| x_24_ | [Bid] | substrate of active caspase-8, inactive form of Bid | 4 × 10^4^ | 4 × 10^2^ | 4 × 10^6^ |
| x_25_ | [C8*:Bid] | compex of active caspase-8 and Bid | 0 | 0 | 0 |
| x_26_ | [tBid] | cleaved Bid, active form of Bid | 0 | 0 | 0 |
| x_27_ | [Bcl-2c] | represents the family of anti-apoptotic Bcl-2 proteins in the cellular compartment (CC), it binds to tBid and acts as inhibitor | 2 × 10^4^ | 2 × 10^2^ | 2 × 10^6^ |
| x_28_ | [tBid:Bcl2c] | complex of cleaved Bid and Bcl2c | 0 | 0 | 0 |
| x_29_ | [Bax] | substrate of tBid, inactive form | 1 × 10^5^ | 1 × 10^3^ | 1 × 10^7^ |
| x_30_ | [tBid:Bax] | complex of cleaved Bid and Bax | 0 | 0 | 0 |
| x_31_ | [Bax*] | active form of Bax | 0 | 0 | 0 |
| x_32_ | [Bax*_m_] | Bax*_m_ in the mitochondrial compartment (MC) | 0 | 0 | 0 |
| x_33_ | [Bcl2] | represents all antiapoptotic Bcl-2 proteins in the MC | 2 × 10^4^ | 2 × 10^2^ | 2 × 10^6^ |
| x_34_ | [Bax*_m_:Bcl2] | complex of Bax*m and Bcl2 | 0 | 0 | 0 |
| x_35_ | [Bax_2_] | represents Bax*_m_:Bax*_m_ (complex of two Bax*_m_) in the MC | 0 | 0 | 0 |
| x_36_ | [Bax_2_:Bcl2] | complex of Bax_2_ and Bcl2 | 0 | 0 | 0 |
| x_37_ | [Bax_4_] | represents Bax2:Bax2 (complex of two Bax_2_) in the MC | 0 | 0 | 0 |
| x_38_ | [Bax_4_:Bcl2] | complex of Bax_4_ and Bcl2 | 0 | 0 | 0 |
| x_39_ | [M] | the number of unoccupied Bcl-2 proteins in the MC | 5 × 10^5^ | 5 × 10^3^ | 5 × 10^7^ |
| x_40_ | [Bax_4_:M] | complex containing Bax_4_ and M | 0 | 0 | 0 |
| x_41_ | [M*] | the number of pores Bax4 created on the outer membrane of the mitochondria | 0 | 0 | 0 |
| x_42_ | [CyC_m_] | cytochrome C inside the mitochondria, in MC | 5 × 10^5^ | 5 × 10^3^ | 5 × 10^7^ |
| x_43_ | [M*:CyC_m_] | complex containing M* and CyC_m_ | 0 | 0 | 0 |
| x_44_ | [CyC_r_] | cytochrome C released from the mitochondria, but remaining in MC | 0 | 0 | 0 |
| x_45_ | [Smac_m_] | Smac/Diablo inside the mitochondria, in MC | 1 × 10^5^ | 1 × 10^3^ | 1 × 10^7^ |
| x_46_ | [M*:Smac_m_] | complex containing M* and Smac_m_ | 0 | 0 | 0 |
| x_47_ | [Smac_r_] | Smac/Diablo released from the mitochondria, but remaining in MC | 0 | 0 | 0 |
| x_48_ | [CyC] | cytochrome c in the CC | 0 | 0 | 0 |
| x_49_ | [Apaf] | Apoptosis Activating Factor (Apaf-1), substrate of CyC, inactive form | 1 × 10^5^ | 1 × 10^3^ | 1 × 10^7^ |
| x_50_ | [Apaf:CyC] | complex containing Apaf and cytochrome c | 0 | 0 | 0 |
| x_51_ | [Apaf*] | active form of Apaf-1 | 0 | 0 | 0 |
| x_52_ | [C9] | procaspase-9 | 1 × 10^5^ | 1 × 10^3^ | 1 × 10^7^ |
| x_53_ | [Apop] | the apoptosome, which is the complex Apaf*:C9 | 0 | 0 | 0 |
| x_54_ | [Apop:C3] | complex containing the apoptosome and procaspase-3 | 0 | 0 | 0 |
| x_55_ | [Smac] | Smac/Diablo in the CC | 0 | 0 | 0 |
| x_56_ | [Apop:XIAP] | complex containing the apoptosome and XIAP | 0 | 0 | 0 |
| x_57_ | [Smac:XIAP] | complex containing Smac and XIAP | 0 | 0 | 0 |
| x_58_ | [C3*_Ub_] | C3* ubiquinated and targeted for degradation, assumed inactive | 0 | 0 | 0 |
