# Supplementary material for: Decision-Tree Based Model Analysis for Efficient Identification of Parameter Relations Leading to Different Signaling States
Source: PLoS One. 2013 Dec 18;8(12):e82593. doi: 10.1371/journal.pone.0082593 (PMC3867358; doi:10.1371/journal.pone.0082593)
Supplement: Text S1 — Additional methods and results for unsupervised clustering of trajectories obtained by simulation on the caspase-3 activation model from 50,000 random parameter sets. (DOCX) [file pone.0082593.s013.docx]

**Supporting Methods and Results**

**Methods**

The model by Aldridge (ref. 3 of the manuscript) has been used to simulate output trajectories from 50,000 random parameter sets that have been obtained as described in the manuscript. We used *k*-means clustering to determine clusters for *k*=2, *k*=3 and *k*=4. The algorithm was started from 10 random cluster centroids, and run for a maximum of 100 iterations each. We used R version 3.0.0 (<http://www.r-project.org>) together with packages cluster (v. 1.14.4) and biganalytics (v. 1.1.1). The stability of the clusters has been determined by bootstrapping with 100 replicates using the package fpc (v. 2.1.5) on a multicore architecture.

**Results**

Stability analysis suggested that *k*=2 yielded the most stable clusters as determined from the mean of the recoverage rate for each cluster. The clustering by *k*-means overlapped to a large extent with the class assignment by pre-defined rules described in the manuscript (Tab. 1, Supporting Figure 6).

***Table 1***: Confusion matrix of results from k-means clustering and those from class assignment by pre-defined rules on 50,000 trajectories obtained by simulation from the model of Aldridge et al. (ref. 3 in manuscript) based on random parameter sets as described in the manuscript.

|  | *Pre-defined rules* | *Apoptosis* | *Survival* |
| --- | --- | --- | --- |
| *k*-means clust. | Apoptosis | 1506 | 1956 |
|  | Survival | 0 | 46538 |

The association between the two methods for class assignment is highly significant (P < 2.2 x 10^–16^ as determined by the chi-square test).
